# Health Care–Associated Infections Among Neonates During the COVID-19 Pandemic

**DOI:** 10.1001/jamanetworkopen.2025.55623

**Published:** 2026-01-28

**Authors:** Sagori Mukhopadhyay, Mark Conaway, Miren B. Dhudasia, Dustin D. Flannery, Michael T. Favara, Pablo J. Sánchez, Jörn-Hendrik Weitkamp, Sarah Khan, Kristin E. D. Weimer, Margaret Gilfillan, Andrew Berenz, Julie Wohrley, Kathryn Ziegler, Robert L. Schelonka, Redjana Carciumaru, Milica Ivanovic, Joseph B. Cantey, Deborah J. Tuttle, Katia C. Halabi, Ryley Guay, Lakshmi Srinivasan, David A. Kaufman

**Affiliations:** 1 Division of Neonatology, Children’s Hospital of Philadelphia, Philadelphia, Pennsylvania; 2 Department of Pediatrics, University of Pennsylvania Perelman School of Medicine, Philadelphia; 3 Department of Public Health Sciences, University of Virginia, Charlottesville; 4 Department of Pediatrics, ChristianaCare, Newark, Delaware; 5 Department of Pediatrics, Division of Neonatology and Pediatric Infectious Diseases, Nationwide Children’s Hospital, The Ohio State University College of Medicine, Columbus; 6 Department of Pediatrics, Vanderbilt University, Nashville, Tennessee; 7 Department of Pediatrics McMaster University, Hamilton, Ontario, Canada; 8 Department of Pediatrics, Duke University, Durham, North Carolina; 9 Department of Pediatrics, St Christopher’s Hospital for Children, Philadelphia, Pennsylvania; 10 Department of Pediatrics, Rush University Medical Center, Chicago, Illinois; 11 Department of Pediatrics, Jefferson University, Philadelphia, Pennsylvania; 12 Department of Pediatrics, Oregon Health & Science University, Portland; 13 Department of Pediatrics, University of Texas Health Sciences Center, San Antonio; 14 Department of Pediatrics, University of Virginia, Charlottesville

## Abstract

**Importance:**

Neonatal intensive care units (NICUs) implemented enhanced infection prevention measures during the COVID-19 pandemic to interrupt viral transmission. It was hypothesized that these measures would also reduce nonviral health care–associated infections (HAIs).

**Objective:**

To compare rates of viral and bacterial or fungal HAIs in infants admitted to the NICU before and during the pandemic.

**Design, Setting, and Participants:**

This cohort study was conducted at 12 level 3 or level 4 NICUs in the US and Canada. Participants were inborn and outborn infants admitted from March 1, 2018, to July 31, 2022, for at least 1 overnight stay at participating NICUs. Data analyses were performed from September 1, 2023, to July 28, 2025.

**Exposure:**

The viral and bacterial or fungal HAI rates during the pandemic (April 1, 2020, to July 31, 2022) were compared with those before the pandemic (March 1, 2018, to March 31, 2020).

**Main Outcomes and Measures:**

HAI incidence before and during the pandemic was expressed as episodes per 1000 patient-days and compared using pre-post and time series analysis with Poisson regression. HAI was defined as an infection diagnosed 3 or more days after admission and was confirmed using molecular diagnostics for viruses and culture of blood, cerebrospinal fluid, or urine for bacteria or fungal pathogens.

**Results:**

The full cohort comprised 48 475 infants, grouped into the viral HAI or bacterial or fungal HAI subpopulations. Among 41 889 infants with 966 025 patient-days admitted at 11 NICUs, 231 (mean [SD] gestational age, 30.5 [5.3] weeks; 147 males [63.6%]) had 241 episodes of viral HAI. Viral HAI rates decreased from 0.35 to 0.16 per 1000 patient-days with an adjusted rate ratio (aRR) of 0.45 (95% CI, 0.34-0.59). Reduced rates persisted during the later pandemic period (April 1, 2021, to July 31, 2022) compared with the corresponding period before the pandemic (aRR, 0.58; 95% CI, 0.42-0.80), even as viral infections in the community increased. Among 48 475 infants with 1 130 038 patient-days at 12 study NICUs, 1537 (mean [SD] gestational age, 29.4 [5.2] weeks; 930 males [60.5%]) had 1969 episodes of bacterial or fungal HAI. Bacterial or fungal HAI rates did not decrease from before to during the pandemic (1.70 to 1.78 per 1000 patient-days; aRR, 1.04; 95% CI, 0.95-1.14). At the site level, changes in bacterial or fungal HAI rates did not correlate with viral HAI rates.

**Conclusions and Relevance:**

This cohort study found that viral HAIs decreased significantly during the pandemic, while bacterial or fungal HAIs did not. These findings suggest that the enhanced infection prevention measures used during the pandemic may be beneficial during periods of high viral activity but offer limited additional benefit for preventing bacterial or fungal HAI.

## Key Points

**Question:**

Did health care–associated infection (HAI) in neonatal intensive care units (NICUs) decrease with enhanced infection prevention measures implemented during the COVID-19 pandemic?

**Findings:**

In this cohort study, among 41 889 infants admitted to 11 NICUs, viral HAI rates significantly decreased and lower rates persisted during the late pandemic period, despite increasing community viral infection rates. Rates of bacterial or fungal HAI among 48 475 infants admitted to 12 NICUs were unchanged and did not correlate with the site-specific change of viral HAI.

**Meaning:**

The findings of this study suggest that enhanced infection prevention measures implemented during the pandemic may be beneficial during a high viral burden season; however, reduction in bacterial or fungal HAI will likely require additional or different interventions.

## Teaser

This cohort study examines the incidence of viral and bacterial or fungal infections in neonatal intensive care units and the role of COVID-19–related infection control and prevention measures in rate reduction.

## Introduction

Neonatal health care–associated infections (HAIs) are a major source of mortality, survivor morbidity, and health care costs.^1^ A substantial portion of HAIs may be preventable with optimal adoption of infection prevention and control (IPC) measures.^2^ During the COVID-19 pandemic, neonatal intensive care units (NICUs) implemented universal masking, increased visitor screening and restrictions, and reinforced established measures such as handwashing.^3,4,5,6^ While these measures aimed to interrupt COVID-19 transmission, they can also reduce transmission of other health care–associated viral, bacterial, and fungal pathogens.^2,3,7,8^ For instance, masking may reduce face and nose touching and may then interrupt *Staphylococcus aureus* transmission.^7,9,10,11^ Unlike adults, NICU patients acquired COVID-19 infection less frequently, allowing the assessment of HAI changes with the enhanced IPC measures without the countering effect of acuity in COVID-19 cases.^12,13,14^ However, few multicenter studies in the US have reported infection rates in the NICU during the pandemic, and those studies that have found conflicting results.^15,16,17,18,19^ Additionally, other than in single-center studies,^15,20,21^ the microbiological origin of HAI is underreported.^16,17,18^ Lastly, few studies have quantified changes in viral infections in the NICU, findings that may inform responses to future periods of high viral burden.^22,23^

This study aimed to compare rates of viral and bacterial or fungal HAIs in infants admitted to the NICU before and during the pandemic. The pandemic-associated IPC measures were hypothesized to reduce both viral and bacterial or fungal HAIs, particularly those caused by *S aureus,* a bacteria capable of airborne transmission and for which spread has been reduced with masking in some settings.^24,25,26,27,28^

## Methods

### Design and Setting

This observational cohort study was set in 12 level 3 and level 4 NICUs in the US and Canada.^29^ Study sites reported a median (IQR) annual admission rate of 800 (600-900). Six sites had an open-bay design, 3 had single-patient rooms, and 3 had a mixed design. One level 4 NICU did not submit viral HAI data, and that site was excluded from the denominator when analyzing viral HAI outcomes. The institutional review board at each site approved this study and waived the informed consent requirement because the study posed minimal risk to participants and used limited EHR based data. We followed the Strengthening the Reporting of Observational Studies in Epidemiology (STROBE) reporting guideline.

### Population and Data Sources

All infants admitted to the NICU from March 1, 2018, to July 31, 2022, with at least 1 overnight stay were included. Demographic and infection-related data for infants with HAI were collected by site-specific research staff. Monthly admissions, mortality, patient-days, and central-line days were collected as aggregate data, stratified by birth weight categories. All data were entered in centralized case-report forms in REDCap.^30^ Race and ethnicity were captured from site-specific electronic medical record, wherein entries primarily reflect patient self-reported information. These data were collected to account for social and structural factors that may influence outcomes between the exposure periods.

### Exposure Periods

Site representatives completed a survey of IPC measures during the pandemic. April 1, 2020, was selected as the start of the pandemic period as all sites had implemented IPC measures by this point, including universal masking, changes to staff and visitor health screening, and restricted visitation policies (eTable 1 in Supplement 1). For diagnostic tests “always or frequently” obtained during HAI evaluation, all sites selected complete blood count, blood culture, and urine culture and half of the sites selected cerebrospinal fluid cultures and respiratory viral panels, both before and during the pandemic (eTable 2 in Supplement 1). Routine addition of respiratory viral panel tests in HAI evaluation was reported by 1 site during the pandemic.

### Outcomes

HAI was defined as an infection diagnosed on or after the third day of admission. It was measured as the rate of infection episodes per 1000 patient-days.^31^

Viral HAI included episodes where a virus was identified. Herpes simplex and cytomegalovirus infections were excluded given that the primary transmission for these viruses was perinatal, congenital, or through mother’s milk, routes not impacted by IPC measures. As many diagnostic tests cannot differentiate rhinovirus from enterovirus, results for these viruses were combined. Repeat detection of the same virus from an infant was considered as a single episode, as virus shedding can be prolonged.^32^ Detection of a different virus in an infant was defined as a new episode.

Bacterial or fungal HAI included episodes where a known pathogen was identified in cultures from blood (bloodstream infection [BSI]), cerebrospinal fluid (meningitis), or urine (urinary tract infection [UTI]) and treated by the clinical team for 5 or more days. Common commensals were excluded as contaminants, including *Micrococcus*, *Propionibacterium*, corynebacteria, *Bacillus*, *Lactobacillus* sp, and diphtheroids. Polymicrobial cultures with at least 1 pathogen were included. Coagulase-negative staphylococci (CONS) were included if the infant was treated for 5 days or more.^19,33^ Due to challenges in distinguishing CONS infection from contamination, CONS were analyzed separately as a pathogen group along with gram-positive (excluding CONS), gram-negative, fungus, and polymicrobial infections. There is a lack of consensus in defining UTI among neonates.^34,35,36,37^ To allow for this, UTI was defined in 2 ways: treated UTIs, including all episodes with 1 or more noncommensal pathogens that the clinician treated for more than 5 days, and confirmed UTIs, including treated UTIs where a single pathogen was identified at more than 10 000 colony-forming units (CFU)/mL. A threshold of 10 000 CFU/mL was selected instead of 50 000 CFU/mL^38^ to accommodate sites that reported ranges of 10 000 to 100 000 CFU/mL.^35,36,37^ Central line–associated bloodstream infection was defined using the 2020 National Healthcare Safety Network criteria,^31^ and incidence was measured per 1000 central-line days. A new bacterial or fungal HAI episode was defined as a new pathogen cultured at any time or a repeat pathogen cultured after 10 days from the previous episode.

### Statistical Analysis

Bivariable analysis was performed using the χ^2^ test, Fisher exact test, and Wilcoxon test as appropriate. A 2-sided *P* < .05 was considered statistically significant. The Mantel-Haenszel test, adjusting for site, was used to compare the proportion of infants with HAI before and during the pandemic.

HAI incidence rates were compared in a pre-post analysis and an interrupted time series (ITS) model using Poisson regression. A rate ratio (RR) with 95% CI was computed for the pre-post analysis and adjusted for site as a random effect. The lower the value of the ratio, the greater the reduction in rates during a pandemic. For the ITS analysis, methods described by Liu et al^39^ were used. The ITS model estimated trends in monthly HAI rates before the pandemic, change in the rate at the time of the interruption (April 1, 2020) as a level change, and the trend thereafter during the pandemic. The change in the level reflected an immediate consequence of the pandemic-related measures, and the trend thereafter reflected the long-term consequence.^40,41^

Community burden of viral infections decreased during the first year of the pandemic but increased in the second year (April 1, 2021, to July 31, 2022), with least fluctuations observed for rhinovirus infection.^42,43^ To assess whether changes in viral HAI in the NICU persisted in these scenarios, we compared the overall and seasonal (April to September and October to March) rates for all viral HAI and for rhinovirus and/or enterovirus HAI in the second pandemic year against matched periods before the pandemic. To assess the role of site compliance, we compared site-specific changes in bacterial or fungal and viral HAIs using Spearman rank correlation, anticipating that they would correlate. A prespecified secondary analysis assessed HAI rates in extremely low-birth-weight (ELBW; birth weight <1 kg) infants. A 2-sided *P* < .05 was considered statistically significant. All analyses were performed from September 1, 2023, to July 28, 2025, using SAS, version 9.4 (SAS Institute).

## Results

The full cohort comprised 48 475 infants, who composed the viral HAI group or bacterial or fungal HAI group. A total of 41 889 infants admitted (966 025 patient-days) to 11 NICUs reported viral HAI. Of these infants, 231 (0.6%) had 241 episodes of viral HAI (eFigure in Supplement 1), with an overall rate of 0.25 (95% CI, 0.22-0.28) per 1000 patient-days. These infants had a mean (SD) birth weight of 1568 (1030) g, had a mean (SD) gestational age of 30.5 (5.3) weeks, and included 83 females (36.1%) and 147 males (63.6%) (Table 1).

**Table 1.** Characteristics of Infants With Viral and Bacterial or Fungal Health Care–Associated Infection Before and During the COVID-19 Pandemic

Characteristic	Infants, No. (%)^a^							
	Viral HAI group (n = 41 889)				Bacterial or fungal HAI group (n = 48 475)			
	Total	Before pandemic	During pandemic	*P* value	Total	Before pandemic	During pandemic	*P* value
No.	231	153	78	NA	1537	724	813	NA
Birth weight, mean (SD), g	1568 (1030)	1513 (1061)	1678 (971)	.24	1387 (942)	1389 (945)	1386 (940)	.94
Gestational age, mean (SD), wk	30.5 (5.3)	30.1 (5.3)	31.1 (5.1)	.16	29.4 (5.2)	29.4 (5.3)	29.4 (5.2)	.82
Race and ethnicity^b^								
Hispanic	31 (13.4)	18 (11.8)	13 (16.7)	.48	148 (9.6)	69 (9.5)	79 (9.7)	.17
Non-Hispanic American Indian or Alaska Native	1 (0.4)	0	1 (1.3)		3 (0.2)	1 (0.1)	2 (0.2)	
Non-Hispanic Asian	14 (6.1)	11 (7.2)	3 (3.9)		42 (2.7)	20 (2.8)	22 (2.7)	
Non-Hispanic Black	65 (28.1)	44 (28.8)	21 (26.9)		495 (32.2)	213 (29.4)	282 (34.7)	
Non-Hispanic Native Hawaiian or Pacific Islander	0 (0)	0 (0)	0 (0)		3 (0.2)	1 (0.1)	2 (0.2)	
Non-Hispanic White	87 (37.7)	56 (36.6)	31 (39.7)		517 (33.6)	241 (33.3)	276 (34.0)	
Non-Hispanic more than 1 race	9 (3.9)	5 (3.3)	4 (5.1)		22 (1.4)	12 (1.7)	10 (1.2)	
Other^c^	1 (0.4)	1 (0.7)	0 (0)		14 (0.9)	6 (0.8)	8 (1.0)	
Missing data	23 (10.0)	18 (11.8)	5 (6.4)		293 (19.1)	161 (22.2)	132 (16.2)	
Sex^d^								
Female	83 (36.1)	52 (34.0)	31 (40.3)	.35	605 (39.4)	282 (39.0)	323 (39.8)	.77
Male	147 (63.6)	101 (66.0)	46 (59.0)		930 (60.5)	441 (60.9)	489 (60.2)	
Multiple gestation	34 (14.7)	19 (12.4)	15 (19.2)	.17	269 (17.5)	138 (19.1)	131 (16.1)	.13
Delivery mode, vaginal^d^	86 (37.2)	57 (37.3)	29 (37.2)	.99	582 (38.0)	285 (39.5)	297 (36.6)	.24
Age at first infection episode, median (IQR), d^e^	95 (41-156)	99 (47-158)	87 (33-150)	.16	26 (12-57)	24 (12-53)	28 (13-59)	.05
No. of infections per infant								
1	203 (87.9)	132 (86.3)	71 (91.0)	.56	1218 (79.2)	564 (77.9)	654 (80.4)	.35
2	21 (9.1)	16 (10.5)	5 (6.4)		243 (15.8)	119 (16.4)	124 (15.3)	
≥3	7 (3.0)	5 (3.3)	2 (2.6)		76 (4.9)	41 (5.7)	35 (4.3)	
Disposition^f^								
Died	12 (5.2)	5 (3.3)	7 (9.2)	.10	216 (14.5)	96 (13.3)	120 (15.6)	.26
Transferred	11 (4.8)	9 (5.9)	2 (2.6)	.34	186 (12.5)	98 (13.6)	88 (11.5)	.75
Discharged home	206 (90.0)	139 (90.9)	67 (88.2)	.60	1087 (73.0)	527 (73.1)	560 (72.9)	.10
Died during HAI event	2 (0.01)	0 (0)	2 (3)	.10	130 (8.5)	63 (8.7)	67 (8.2)	.75

Abbreviations: HAI, health care–associated infection; NA, not applicable.

^a^ There were 23 infants with bacterial or fungal HAI episodes both before and during the pandemic. For this table, these infants were combined with the 701 infants with episodes before the pandemic only. No infants experienced viral HAI in both periods.

^b^ Race and ethnicity were captured from site-specific electronic medical records with primarily self-reported entries.

^c^ Other includes race or ethnicity outside the categories or reported as unspecified.

^d^ In the viral infection group, sex was missing for 1 infant during the pandemic. In the bacterial or fungal infection group, sex was missing for 2 infants (1 from each period). Delivery mode was missing for 4 infants (3 before and 1 during the pandemic).

^e^ *P* value was calculated using Wilcoxon test.

^f^ Disposition analysis for viral infection excluded 2 infants still admitted at the time of data entry. For infants with bacterial or fungal events, disposition analysis excluded 6 infants with missing data and 42 infants still admitted at the time of data entry. *P* value was generated with Fisher exact test.

A total of 48 475 infants admitted (1 130 038 patient-days) across the 12 study sites reported bacterial or fungal HAI. Of these infants, 1537 (3.2%) had 1969 episodes of bacterial or fungal HAI (eFigure in Supplement 1), with an overall rate of 1.74 (95% CI, 1.67-1.82) per 1000 patient-days. These infants had a mean (SD) birth weight of 1387 (942) g, had a mean (SD) gestational age of 29.4 (5.2) weeks, and included 605 females (39.4%) and 930 males (60.5%) (Table 1).

The mean (SD) monthly admission rate per site before vs during the pandemic was not different (75.3 [31.8] vs 77.0 [34.1]; *P* = .51) (eTable 3 in Supplement 1), and neither were the rates of patient-days, line-days, transfer, or mortality. The characteristics of infants with viral HAI or bacterial or fungal HAI did not change significantly during the pandemic (Table 1).

### Viral HAI

**Figure 1.**
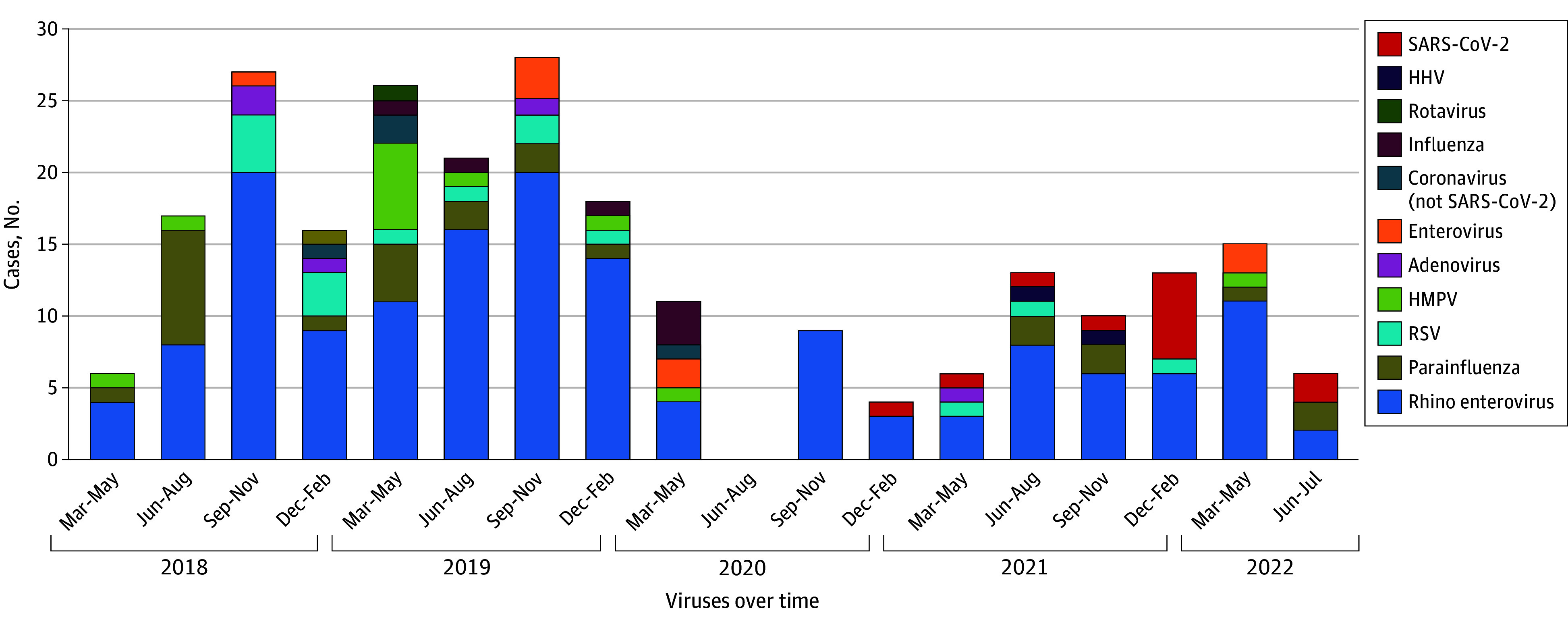
Viruses Identified Before and During the COVID-19 Pandemic HHV indicates human herpesvirus; HMPV, human metapneumovirus; and RSV, respiratory syncytial virus.

Among the 241 viral HAI episodes, 229 (95.0%) were identified on nasopharyngeal swabs (eFigure in Supplement 1). Rhinovirus and/or enterovirus were the most frequent infections both before (104 of 165 [63.0%]) and during (50 of 81 [61.7%]) the pandemic, followed by parainfluenza virus before the pandemic (19 [11.5%]) and SARS-CoV-2 during the pandemic (12 [14.8%]) (Figure 1; eTable 4 in Supplement 1).

**Figure 2.**
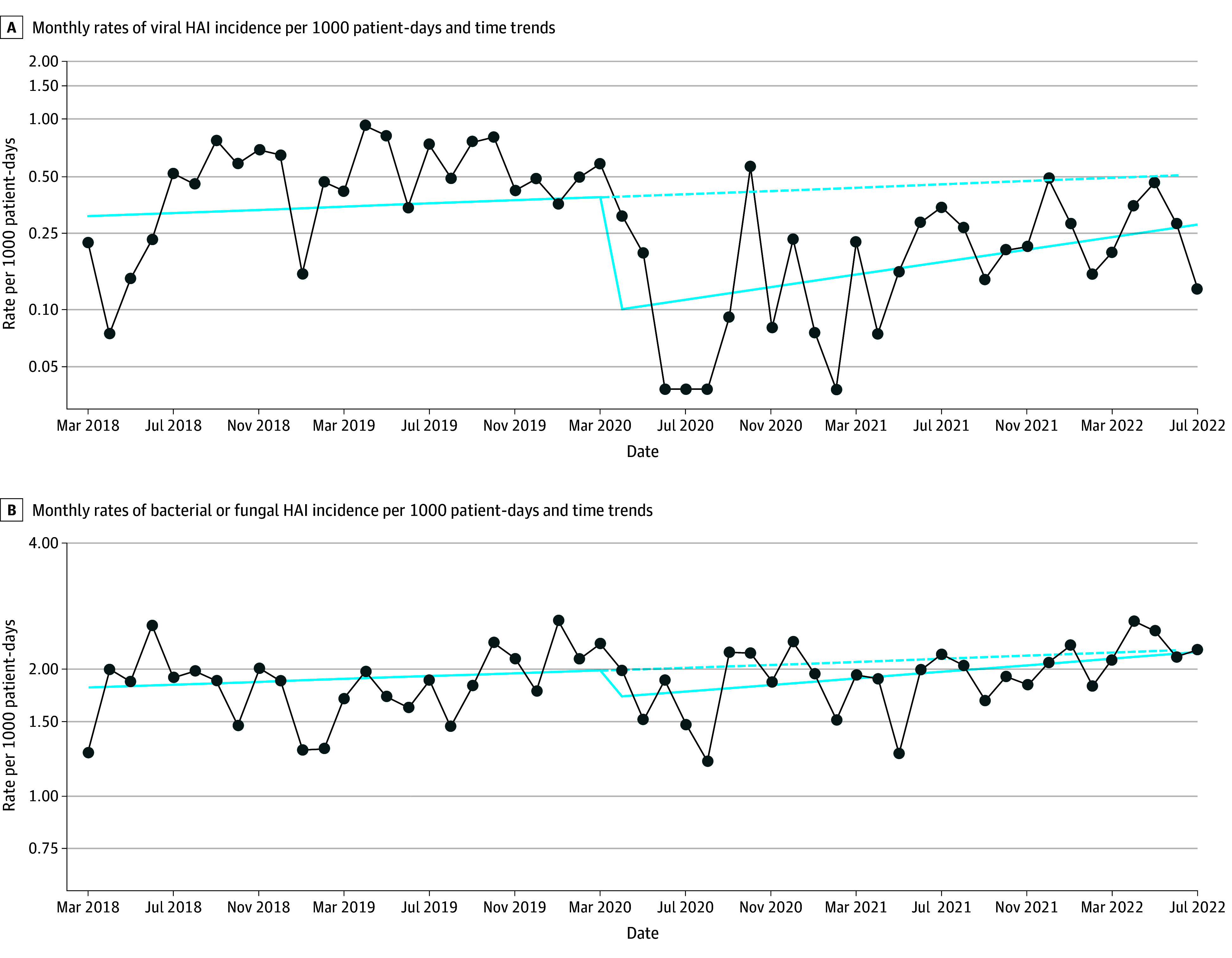
Monthly Health Care–Associated Infection (HAI) Rates and Time Trends Dark blue line indicates actual rates, light blue solid line indicates estimated overall mean monthly rate using the random intercept model fitted to a linear model (as described by Liu et al)^39^ and the light blue dashed line indicates projection of the mean rate from before the COVID-19 pandemic.

The proportion of infants with viral HAI decreased from 0.8% (153) before to 0.3% (72) during the pandemic (*P* < .001) (eTable 5 in Supplement 1). Viral HAI rates per 1000 patient-days decreased from 0.35 (95% CI, 0.28-0.41) to 0.16 (95% CI, 0.13-0.20), with an adjusted RR (aRR) of 0.45 (95% CI, 0.34-0.59) (Table 2). Rates per 1000 patient-days also decreased among ELBW infants (aRR, 0.32; 95% CI, 0.20-0.50). The reduced rate was reflected in the ITS analysis as a reduction in level at the start of the pandemic estimated at −1.41 (95% CI, −2.13 to −0.69) (eTable 6 in Supplement 1; Figure 2A). After the initial reduction, the rate trend increased (0.038; 95% CI, 0.009-0.067), although the overall rates remained below the rates projected before the pandemic (Figure 2A).

**Table 2.** Health Care–Associated Infection Incidence Rates per 1000 Patient-Days Before and During the COVID-19 Pandemic

Characteristic	HAI episodes (rate per 1000 patient-days)			aRR (95% CI)^a^	*P* value
	Total	Before pandemic	During pandemic		
Patient-days at 11 sites, No.	966 025	464 657	501 368	NA	NA
Viral infection episodes	241 (0.25)	162 (0.35)	79 (0.16)	0.45 (0.34-0.59)	<.001
ELBW infants, patient-days, No.	315 336	156 369	158 967	NA	NA
Viral infection episodes	103 (0.33)	78 (0.50)	25 (0.16)	0.31 (0.20-0.49)	<.001
Patient-days at 12 sites, No.	1 130 038	535 055	594 983	NA	NA
Bacterial or fungal infection episodes	1969 (1.74)	912 (1.70)	1057 (1.78)	1.04 (0.95-1.14)	.37
Type of infections^b^					
Any BSI	1058 (0.94)	504 (0.94)	554 (0.93)	0.99 (0.88-1.11)	.90
Any meningitis	41 (0.04)	22 (0.04)	19 (0.03)	0.77 (0.42-1.43)	.41
BSI and meningitis	1086 (0.96)	520 (0.97)	566 (0.95)	0.98 (0.87-1.10)	.72
Treated UTI alone	883 (0.78)	392 (0.73)	491 (0.83)	1.12 (0.98-1.28)	.08
Confirmed UTI alone	551 (0.49)	245 (0.46)	306 (0.51)	1.12 (0.94-1.32)	.19
CLABSI, No. (per 1000 line-days)^c^	235 (0.99)	109 (0.93)	126 (1.05)	1.13 (0.87-1.46)	.36
BSI and/or meningitis by pathogen group					
CONS	439 (0.39)	227 (0.42)	212 (0.36)	0.84 (0.69-1.01)	.06
Gram-positive, excluding CONS	273 (0.24)	112 (0.21)	161 (0.27)	1.29 (1.02-1.65)	.04
Gram-negative	273 (0.24)	139 (0.26)	134 (0.23)	0.87 (0.68-1.10)	.24
Fungal	22 (0.02)	9 (0.02)	13 (0.02)	1.31 (0.56-3.06)	.54
Polymicrobial^d^	79 (0.07)	33 (0.06)	46 (0.08)	1.25 (0.80-1.96)	.32
Treated UTI by pathogen group					
CONS	35 (0.03)	19 (0.04)	16 (0.03)	0.76 (0.39-1.48)	.42
Gram-positive, excluding CONS	136 (0.12)	59 (0.11)	77 (0.13)	1.17 (0.83-1.65)	.36
Gram-negative	512 (0.45)	231 (0.43)	281 (0.47)	1.09 (0.91-1.29)	.35
Fungal	28 (0.02)	16 (0.03)	12 (0.02)	0.67 (0.32-1.42)	.30
Polymicrobial^d^	172 (0.15)	67 (0.13)	105 (0.18)	1.42 (1.04-1.92)	.03
Confirmed UTI by pathogen group					
CONS	22 (0.02)	12 (0.02)	10 (0.02)	0.75 (0.33-1.75)	.51
Gram-positive, excluding CONS	92 (0.08)	40 (0.07)	52 (0.09)	1.17 (0.77-1.76)	.46
Gram-negative	418 (0.37)	181 (0.34)	237 (0.40)	1.17 (0.97-1.42)	.11
Fungal	19 (0.02)	12 (0.02)	7 (0.01)	0.52 (0.21-1.33)	.17
ELBW infants, patient-days, No.	366 733	176 173	190 560	NA	NA
ELBW infants, bacterial or fungal infection episodes	1055 (2.88)	482 (2.74)	573 (3.01)	1.1 (0.98-1.25)	.11

Abbreviations: aRR, adjusted rate ratio; BSI, bloodstream infection; CLABSI, central line–associated bloodstream infection; CONS, coagulase-negative staphylococci; ELBW, extremely low birth weight; HAI, health care–associated infection; NA, not applicable; UTI, urinary tract infection.

^a^ Calculation was adjusted for site. Rate change is significant if the 95% CI does not include 1.

^b^ Any BSI or any meningitis includes episodes of BSI and/or meningitis (with or without concomitant infection with another source). Of the 1058 BSI episodes, 988 were BSIs alone, 57 were concomitant UTIs, 1 was concomitant UTI and meningitis, and 12 were concomitant meningitis. Of the 41 meningitis episodes, 23 were meningitis alone, 12 were concomitant BSIs, 5 were concomitant UTIs, and 1 was concomitant BSI and UTI. UTI-alone episodes include infants whose UTI was not associated with any other source of infection, such that treatment could be attributed to it.

^c^ CLABSI information was missing for 6 BSI episodes. Number of line-days was 117 101 for before the pandemic and 120 227 for during the pandemic.

^d^ If more than 1 organism was isolated from the same specimen on the same day, it was considered a polymicrobial infection and the proportion of such infections is shown. Organisms in these episodes were not included in the pathogen group calculations. A list of all organisms, including those identified in polymicrobial infections, can be found in eTable 8 in Supplement 1.

In the second year of the pandemic (April 1, 2021, to July 31, 2022), rates per 1000 patient-days remained lower than before (March 1, 2018, to March 31, 2020) the pandemic (aRR, 0.58; 95% CI, 0.42-0.80), both overall and across seasons (eTable 7 in Supplement 1). Rates of rhinovirus and/or enterovirus per 1000 patient-days also remained lower in the second year compared with rates before the pandemic (aRR, 0.59; 95% CI, 0.38-0.89).

### Bacterial or Fungal HAI

Of the 1969 episodes of bacterial or fungal HAI, 988 were BSIs, 23 were meningitis, and 883 were UTIs. The remaining 75 episodes involved multiple sources: BSI and meningitis (n = 12), BSI or UTI (n = 57), meningitis or UTI (n = 5), and all 3 types (n = 1) (eFigure in Supplement 1). Rates per 1000 patient-days of BSI and/or meningitis were 0.96 (95% CI, 0.91-1.02) among all admissions, and 1.72 (95% CI, 1.59-1.86) among ELBW infants. Rates of treated UTI per 1000 patient-days were 0.78 (95% CI, 0.73-0.84) and 1.15 (95% CI, 1.05-1.27) among ELBW infants.

In the 1086 BSI and/or meningitis episodes, 1175 organisms were identified, including 79 polymicrobial episodes (7.3%) (eTable 8 in Supplement 1). CONS were the most frequent (528 [44.9%]), followed by *S aureus* (173 [14.7%]) and *E coli* (145 [12.3%]). In the 883 episodes of treated UTI, 1070 organisms were identified, including 172 polymicrobial episodes (19.5%) (eTable 8 in Supplement 1). Gram-negative organisms were the most frequent (720 [67.3%]), specifically *Klebsiella* sp (273 [25.5%]), followed by gram-positive organisms (320 [29.9%]), most frequently *Enterococcus* sp (218 [20.4%]).

The proportion of infants with bacterial or fungal HAI did not change before vs during the pandemic (3.1% [701] vs 3.0% [785]; *P* = .41) (eTable 5 in Supplement 1). Rates per 1000 patient-days were also unchanged between these periods, ranging from 1.70 to 1.78 (aRR, 1.04; 95% CI, 0.95-1.14) (Table 2). Among pathogen groups, rates of *S aureus* did not decrease (aRR, 1.17; 95% CI, 0.89-1.55), while rates of BSI and/or meningitis from non-CONS gram-positive pathogens (aRR, 1.29; 95% CI, 1.02-1.65) and polymicrobial UTI increased (aRR, 1.42; 95% CI, 1.04-1.92) during the pandemic. Among ELBW infants, there was a significant increase in rates of treated UTI (aRR, 1.30; 95% CI, 1.07-1.58) and polymicrobial UTI (aRR, 1.99; 95% CI, 1.56-3.17) (eTable 9 in Supplement 1). Rates of confirmed UTI were not different overall or among ELBW infants.

In the ITS analysis, HAI rates per 1000 patient-days did not change significantly at the start of the pandemic, and the rate trend showed an increase (0.009; 95% CI, 0.001-0.017) during the pandemic that was not significantly different from the trend before the pandemic (Figure 2B; eTable 6 in Supplement 1).

### Site-Specific Changes in Viral and Bacterial or Fungal HAI Rates

**Figure 3.**
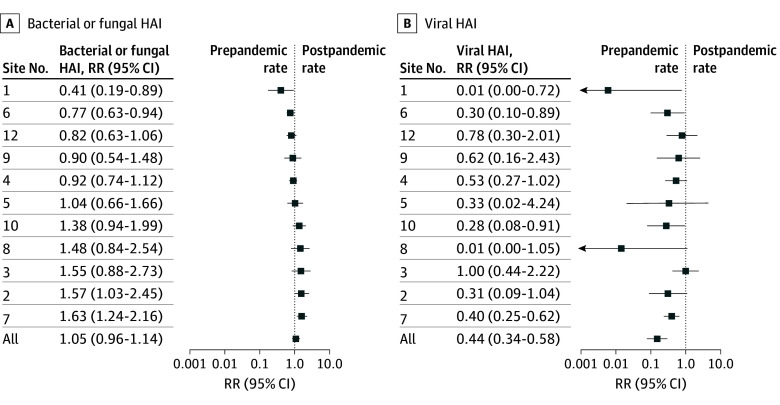
Site Rates of Viral and Bacterial or Fungal Health Care–Associated Infection (HAI) The Spearman rank correlation between bacterial and viral risk ratios was 0.16 (95% CI, −0.57 to 0.62; *P* = .75). RR indicates rate ratio.

Before the pandemic, viral HAI rates at all sites were less than 1 (range, 0.08-0.65) per 1000 patient-days. Bacterial or fungal HAI rates were more varied, from 0.51 to 5.4 per 1000 patient-days. Viral HAI reduced significantly in 4 of 11 sites and did not increase for any site (Figure 3). In contrast, bacterial or fungal HAI rates increased in 2 sites, decreased in 2 sites, and were unchanged in 8 sites. There was no statistically significant correlation with the site-specific viral rates. The Spearman rank correlation between bacterial and viral risk ratios was 0.16 (95% CI, −0.57 to 0.62; *P* = .75).

## Discussion

In this multicenter cohort study of more than 40 000 NICU admissions, the incidence rates of viral HAI decreased significantly during the COVID-19 pandemic. This change was similar across most sites and persisted in the second year of the pandemic. In contrast, overall rates of bacterial or fungal HAI remained largely unchanged. The site-specific magnitude of viral HAI reduction did not correlate with changes in bacterial or fungal HAI rates, making it less likely that site-specific compliance with IPC measures explains the lack of change in bacterial or fungal HAI.

While studies in adult and pediatric settings reported lower viral HAI rates during the pandemic,^44,45^ few reports focused on the NICU. In a single-center surveillance study in Spain, a reduction in positive respiratory viral test results from 8% to 3% was reported in infants with less than 32 weeks’ gestation.^23^ Another study from Western Australia noted a significant decrease in viral respiratory infections during the pandemic without a concomitant reduction in nonrespiratory viral infections.^22^ Distinguishing the role of reduced community viral burden from that of unit IPC measures in viral HAI reduction is challenging.^42,43,46^ To address this question, viral HAI rates were assessed in the second pandemic year, when community viral burden had increased, including rhinovirus, whose community burden was less affected than other respiratory viruses.^42,43,46,47^ Reduction in viral HAI rates, including rhinovirus and/or enterovirus infections, persisted in the second year, supporting a role for the pandemic-related IPC measures in the observed viral HAI rate reduction. Other studies have also reported an independent contribution of the hospital measures.^45,48^ One study found that universal masking and visitor screening were associated with approximately 50% decrease in influenza and respiratory syncytial virus HAI after accounting for community viral burden.^48^ Additionally, in prepandemic studies without community viral fluctuations, IPC measures such as masking^7^ and visitation restrictions^49,50^ were effective in reducing viral infection transmission. Taken together, it is likely that unit-specific IPC measures contributed to the observed decrease in viral HAI.

Nationwide, bacterial or fungal HAIs increased during the pandemic, partly because of the high acuity of patients with COVID-19 infection and partly due to stretched resources.^12,51,52^ However, studies in the NICU have found varying associations, with some reporting decreased incidence^17,18^ or reduced gram-negative HAI^20,21^ and some reporting an increased incidence.^15^ One explanation for this variation could be site differences in adherence to IPC measures. To assess for adherence, we compared changes in viral HAI with those in bacterial or fungal HAI, anticipating that a reduction in one would imply adherence and correlate with a reduction in the other. However, no correlation was found (Figure 3), suggesting that adherence was not a main factor in the unchanged bacterial or fungal HAI rates. It was hypothesized that universal masking would decrease transmission of *S aureus* by limiting transfer from caregiver nasal carriage to their hands and by decreasing aerosolized spread.^26,27,28,53^ This association was not observed. Instead, an increase in non-CONS gram-positive BSI and/or meningitis was identified (Table 2). While this observation may be due to chance, higher rates of *S aureus*, CONS, and enterococci have been reported in health care settings, including the NICU, during the pandemic.^15,51,54^ High rates of mask contamination, specifically with *S aureus*, and increased *S aureus* burden in the nasal microbiome with prolonged masking were described and may counter the benefits of interrupted transmission.^55,56,57,58^ While reducing colonization burden has been proven to decrease *S aureus* infections,^59^ further investigations are needed to understand the pros and cons of masking for this indication.

A secondary analysis restricted to ELBW infants was prespecified due to their risk for HAI. Similar to prior reports, no changes were found in the overall incidence of BSI and/or meningitis among ELBW infants.^19^ In contrast, increased rates of treated UTI, particularly polymicrobial UTI, was observed during the pandemic. A third of these episodes did not meet the criteria for confirmed UTI, rates for which remained unchanged. There is a lack of consensus in defining neonatal UTI particularly in preterm infants.^34,36,38,60^ The findings suggest that the observed increase in UTI may potentially be associated with decreased thresholds for treating UTI among ELBW infants, rather than changes in incidence or pathogen distribution, identifying the need for standardized UTI diagnosis as an important area for antibiotic stewardship in the NICU.

### Strengths and Limitations

A strength of the study was the large sample size across multiple sites, including major infection types and linked microbiological information. The study also had several limitations. Detailed patient-level data were collected on HAI cases only; all other admission data were captured as monthly aggregate data. While sites served as their own control and monthly rates of overall admissions and admission in different birth weight categories remained stable (eTable 3 in Supplement 1), lack of patient-level data for all admissions precluded multivariate risk adjustment. Site surveys identified the timing of IPC measures, but information on adherence was not available. Standard definitions were used to identify HAI cases, but data on urine collection techniques, urinalysis results, and other clinical criteria were unavailable. Several IPC measures changed (eTable 1 in Supplement 1) concurrently, the findings reflect the combined association of these interventions with outcomes. Although masking was a major component of the IPC measures during the pandemic in the study sites and is known to reduce droplet transmission, other measures, such as visitation restriction, may have also played a role.^61^ Future considerations when implementing universal masking also required attention to unintended consequences, including alteration of the nonviral microbiome^56^ and barriers to communication.^62^ Recent guidance from the Society for Healthcare Epidemiology of America on strategies to prevent viral HAI in the NICU recommends routine masking for clinicians only during periods of high viral transmission or outbreaks.^63^ Identifying the threshold of community or unit transmission at which masking and other measures should be introduced remains an important area of ongoing research.^64^

## Conclusions

In this multicenter cohort study of infants admitted to the NICU, a significant decrease in viral HAIs was observed during the COVID-19 pandemic, indicating the potential utility of these measures in seasons with a high viral disease burden. However, preventive interventions beyond those implemented during the pandemic would likely be necessary to further reduce bacterial or fungal HAIs.

## Notes

Supplement 1. **eFigure.** Infants and HAI Episodes Eligible for Inclusion **eTable 1.** Infection Prevention Practices in Study Sites Before and During Pandemic **eTable 2.** Infection Evaluation and Management Practices After 3 Days of Admission at Study Sites Before and After the Pandemic **eTable 3.** Mean Monthly Admission Measures per Site by Birthweight Category **eTable 4.** Organism Distribution of Viral HAI Events Before and During the Pandemic **eTable 5.** HAI Incidence Proportion Before and During the Pandemic **eTable 6.** Estimates (95% Confidence Limits) of Changes in HAI Rates per 1000 Patient-Days From the Time Series Analyses **eTable 7.** Rate Ratios of Viral HAI the First and Second Year of the Pandemic Compared to Corresponding Periods Before the Pandemic by Seasons **eTable 8.** Organism Distribution in Bacterial/Fungal HAI Before and During the Pandemic **eTable 9.** Bacterial/Fungal HAI Incidence Rates per 1000 Patient-Days Among Extremely Low Birth Weight Infants

Supplement 2.
Data Sharing Statement
