# Supplementary material for: Health Care–Associated Infections Among Neonates During the COVID-19 Pandemic
Source: JAMA Netw Open. 2026 Jan 28;9(1):e2555623. doi: 10.1001/jamanetworkopen.2025.55623 (PMC12853212; doi:10.1001/jamanetworkopen.2025.55623)
Supplement: Supplement 1. — eFigure. Infants and HAI Episodes Eligible for Inclusion eTable 1. Infection Prevention Practices in Study Sites Before and During Pandemic eTable 2. Infection Evaluation and Management Practices After 3 Days of Admission at Study Sites Before and After the Pandemic eTable 3. Mean Monthly Admission Measures per Site by Birthweight Category eTable 4. Organism Distribution of Viral HAI Events Before and During the Pandemic eTable 5. HAI Incidence Proportion Before and During the Pandemic eTable 6. Estimates (95% Confidence Limits) of Changes in HAI Rates per 1000 Patient-Days From the Time Series Analyses eTable 7. Rate Ratios of Viral HAI the First and Second Year of the Pandemic Compared to Corresponding Periods Before the Pandemic by Seasons eTable 8. Organism Distribution in Bacterial/Fungal HAI Before and During the Pandemic eTable 9. Bacterial/Fungal HAI Incidence Rates per 1000 Patient-Days Among Extremely Low Birth Weight Infants [file jamanetwopen-e2555623-s001.pdf]

Supplemental Online Content

Mukhopadhyay S, Conaway M, Dhudasia MB, et al. Health care–associated infections among neonates during the COVID-19 pandemic. *JAMA Netw Open*. 2026;9(1):e2555623. doi:10.1001/jamanetworkopen.2025.55623

eFigure. Infants and HAI Episodes Eligible for Inclusion

eTable 1. Infection Prevention Practices at Study Sites Before and During Pandemic

eTable 2. Infection Evaluation and Management Practices After 3 Days of Admission at Study Sites Before and After the Pandemic

eTable 3. Mean Monthly Admission Measures per Site by Birthweight Category

eTable 4. Organism Distribution of Viral HAI Events Before and During the Pandemic

eTable 5. HAI Incidence Proportion Before and During the Pandemic

eTable 6. Estimates (95% Confidence Limits) of Changes in HAI Rates per 1000 Patient-Days From the Time Series Analyses

eTable 7. Rate Ratios of Viral HAI the First and Second Year of the Pandemic Compared to Corresponding Periods Before the Pandemic by Seasons

eTable 8. Organism Distribution in Bacterial/Fungal HAI Before and During the Pandemic

eTable 9. Bacterial/Fungal HAI Incidence Rates per 1000 Patient-Days Among Extremely Low Birth Weight Infants

This supplemental material has been provided by the authors to give readers additional information about their work.

eFigure 1: Infants and HAI episodes eligible for inclusion.

Figure Legend: [1]Among the 75 bacterial/fungal infections where pathogens were identified from more than one source, blood with urine was most common (57), followed by blood with CSF (12), CSF with urine (5) and blood with CSF and urine (1). [2]The three viral episodes where the pathogen was identified from more than one source included: nasopharyngeal with CSF (2), nasopharyngeal with blood (1). The 12 viral episodes in sources other than nasopharyngeal specimens were: CSF (6), Blood (1), Tracheal Aspirate/bronchoalveolar lavage (4) stool (1).

eTable 1: Infection prevention and control practices at study sites before and during pandemic

Infection prevention practice	Baseline practice	Sites that changed practice (%)	Salient changes
Masking	No routine masking	12 (100)	- Masking for providers and families (7 sites)<br>- Masking only for providers (5 sites)[1]
Visitation	Who can enter?<br>- Legal guardians and family (12 sites)<br>- Excluding young children during flu season (10 sites)<br>Timing<br>- -Anytime for parents (12 sites)<br>- -Limited for visitors (3 sites)<br>How many can be at bedside?<br>- 1-2 persons (8 sites)<br>- >2 persons (4 sites)	12 (100)	Who can enter?<br>- 2 designated legal guardians (11 sites)<br>- Only mother (2 sites)<br>Timing<br>- Anytime (5 sites)<br>- Designated times (7 sites)<br>How many can be at bedside?<br>- 1-2 persons (6 sites)<br>- Only one person (6 sites)
Health screening	General information provided by units advising not to visit unit if febrile or with symptoms of infectious illness.	12 (100)	- Symptom checklist filled by all persons entering unit (11 sites)<br>- Thermal checks for all persons entering the unit (8 sites)<br>- Reporting contact with someone who is ill (11 sites)
Gowning and gloving	Gowning for contact precautions (12 sites)<br>Gloving for contact precautions and high-risk patients such as extremely preterm infants (8 sites); for all patients (1 sites); only in scenarios with potential for contact with bodily fluids (3 sites)	1 (8)	- Universal gloving (1 site)[2]
Admissions and bed allotment criteria[3]	All sites accept potentially infected cases	3 (25)	Admissions potentially reduced due to:<br>- Diversion of admissions from the emergency room (1 site)<br>- COVID-19 exposed neonates admitted to other units in the hospital (1 site)<br>- Reduction in bed capacity to allow social distancing (1 site)

Others	--	4 (33)	- Use of Ultraviolet ray based cleaning equipment (2 sites)<br>- Use of HEPA filters (2 sites)

Footnotes: All changes occurred in March to April of 2020 apart from 1 site where the Ultraviolet ray based cleaning equipment was introduced in August of 2021. All sites had new policies for managing admissions based on their exposure to COVID-19. Listed changes are those that impact all admissions regardless of exposure to COVID-19.
[1]Some policies may have changed over time
[2]Unrelated to COVID-19 changes but coincidental; While gowning and gloving practices did not change, attention to limit excess use, given shortages, was noted by 3 sites.
[3]These changes are outside the more common changes in bed allotment within the unit to allow isolation and cohorting of COVID-19 exposed infants.

Abbreviations: HEPA - High efficiency particulate air

eTable 2: Infection evaluation and management practices after 3 days of admission at study sites before and during the pandemic.

Practice	Before pandemic	During Pandemic
**Routine infection evaluation 'always or frequently' includes**		
Complete blood count assessment	12	12
C-reactive protein assessment	9	8
Other host response biomarker assessment (e.g. procalcitonin)	1	1
**Microbiological tests done 'always or frequently' during evaluation**		
Blood culture	12	12
Urine culture	12	12
Cerebrospinal fluid culture/cell count	5	5
Respiratory viral panel	6	7
SARS-CoV-2 testing	NA	8
**First-line empiric therapies chosen 'always or frequently' for suspected healthcare associated infection**		
Vancomycin	7	7
Gentamicin	11	11
Oxacillin/nafcillin	5	5
Cephalosporin (3rd/4th generation)	4	4
Other antibiotics as first-line empiric therapy	3	3
**Other NICU policies**		
Fluconazole prophylaxis	10	10

Donor milk	12	12
Human-milk based fortifier	10	10
Probiotics	3	3

eTable 3: Mean monthly admission measures per site by birthweight category

	Before Pandemic	During Pandemic	p-value
**Months x sites**	**300**	**336**	
**NICU admissions, n infants**	**22,593**	**25,882**	**--**
Infants admitted by birthweight category, n (% admissions)			
<750 grams	985 (4.4%)	1,047 (4.0%)	0.09
750 to <1000 grams	995 (4.4%)	1,054 (4.1%)	0.07
1000 to <1500 grams	1,848 (8.2%)	2,015 (7.8%)	0.11
1500 to <2500 grams	6,341 (28.1%)	7,412 (28.6%)	0.16
≥2500 grams	12,424 (55.0%)	14,354 (55.5%)	0.30
Mean admissions per month per site, (SD)	75.3 (31.8)	77.0 (34.1)	0.51
<750 grams	3.3 (2.7)	3.1 (2.6)	0.43
750 to <1000 grams	3.3 (3.1)	3.1 (2.5)	0.42
1000 to <1500 grams	6.2 (3.9)	6.0 (4.0)	0.60
1500 to <2500 grams	21.1 (9.4)	22.1 (11.2)	0.26
≥2500 grams	41.4 (19.4)	42.7 (19.9)	0.40
Mortality, n infants (% admissions)	644 (2.9%)	714 (2.8%)	0.54
Mean mortality per month per site, (SD)	2.2 (2.2)	2.1 (2.3)	0.90
<750 grams	0.8 (1.1)	0.7 (1.0)	0.31
750 to <1000 grams	0.2 (0.5)	0.2 (0.5)	0.48
1000 to <1500 grams	0.2 (0.5)	0.2 (0.5)	0.95
1500 to <2500 grams	0.5 (0.8)	0.4 (0.8)	0.59
≥2500 grams	0.4 (0.8)	0.6 (0.9)	0.07
Transfers, n infants (% admissions)	5,436 (24.1%)	6,099 (23.6%)	0.20
Mean transfers per month per site, (SD)	18.1 (28.8)	18.2 (28.3)	0.99
<750 grams	0.6 (1.2)	0.6 (1.0)	0.31
750 to <1000 grams	0.9 (2.0)	0.7 (1.5)	0.29
1000 to <1500 grams	1.6 (3.4)	1.5 (3.1)	0.70

1500 to <2500 grams	4.8 (8.8)	5.3 (10.1)	0.50
≥2500 grams	10.3 (16.0)	10.1 (15.0)	0.91
**Total Patient days[1]**	**535,055**	**594,983**	**--**
Mean patient-days per month per site, (SD)	1,783.5 (1462.6)	1,770.8 (1412.6)	0.91
<750 grams	318.4 (270.1)	298.1 (267.2)	0.99
750 to <1000 grams	268.9 (257.7)	269.0 (219.5)	0.99
1000 to <1500 grams	342.7 (305.5)	329.7 (268.6)	0.57
1500 to <2500 grams	474.4 (414.6)	477.4 (423.2)	0.93
≥2500 grams	379.2 (311.1)	396.5 (326.9)	0.50
**Total Line-days[1]**	**117,101**	**120,227**	**--**
Mean line-days per month per site, (SD)	390.3 (307.6)	357.8 (288.0)	0.17
<750 grams	96.3 (92.8)	76.4 (87.2)	0.005
750 to <1000 grams	60.0 (60.3)	57.6 (60.1)	0.61
1000 to <1500 grams	62.3 (57.3)	54.1 (48.7)	0.05
1500 to <2500 grams	66.8 (66.0)	64.0 (65.9)	0.59
≥2500 grams	104.9 (115.2)	105.8 (115.5)	0.92

Footnote: [1]Patient days and Line days for July 2021 imputed for one site using mean value across data in July across other years for that site.

eTable 4: Organism distribution of viral HAI events before and during the pandemic

Organism, n (column%)	Total n=246	Before pandemic n=165	During pandemic n=81
Rhinovirus/enterovirus	154 (62.6)	104 (63.0)	50 (61.7)
Parainfluenza	26 (10.6)	19 (11.5)	7 (8.6)
Respiratory syncytial virus	15 (6.1)	12 (7.3)	3 (3.7)
Human metapneumovirus	12 (4.9)	11 (6.7)	1 (1.2)
SARS-CoV-2	12 (4.9)	0 (0)	12 (14.8)
Enterovirus	8 (3.3)	4 (2.4)	4 (4.9)
Coronavirus (not SARS-CoV-2)	6 (2.4)	3 (1.8)	3 (3.7)
Influenza	6 (2.4)	6 (3.6)	0 (0)
Adenovirus	5 (2.0)	4 (2.4)	1 (1.2)
Human herpes virus	1 (0.4)	1 (0.6)	0 (0)
Rotavirus	1 (0.4)	1 (0.6)	0 (0)

Footnote: In five episodes, more than one virus was identified – four with parainfluenza and rhinovirus/enterovirus, and one with coronavirus (not SARS-CoV-2) and rhinovirus/enterovirus.

eTable 5: HAI incidence proportion before and during the pandemic

**Characteristics** *No. of infants (% of all admissions)*	**Total**	**Before pandemic**	**During pandemic**	**P value[1]**
**Infants admitted to the 11 NICUs[2], n**	**41883**	**19562**	**22321**	**-**
Infants with viral HAI	225 (0.54)	153 (0.78)	72 (0.32)	<0.001
**Infants admitted to the 12 NICUs[2], n**	**48424**	**22593**	**25831**	
Infants with bacterial/fungal HAI[3]	1486 (3.07)	701 (3.10)	785 (3.04)	0.41
Type of infections[4]				
BSI	940 (1.94)	446 (1.97)	494 (1.91)	0.52
Meningitis	34 (0.11)	18 (0.12)	16 (0.10)	0.87
UTI (treated) alone	687 (1.46)	322 (1.52)	365 (1.41)	0.52
Infants with CLABSI[5]	220 (0.45)	102 (0.45)	118 (0.46)	0.82
BSI and/or meningitis by pathogen group				
CoNS	400 (0.83)	203 (0.90)	197 (0.76)	0.07
Gram-positive bacteria (excluding CONS)	254(0.52)	105 (0.46)	149 (0.58)	0.05
Gram-negative bacteria	255 (0.53)	130 (0.58)	125 (0.48)	0.16
Fungal	20 (0.06)	8 (0.05)	12 (0.06)	0.57
Polymicrobial[6]	76 (0.21)	32 (0.18)	44 (0.23)	0.53
UTI (treated) by pathogen group				
CoNS	34 (0.12)	18 (0.16)	16 (0.10)	0.23
Gram-positive bacteria (excluding CONS)	126 (0.29)	57 (0.30)	69 (0.28)	0.82
Gram-negative bacteria	416 (0.91)	198 (0.93)	218 (0.89)	0.55
Fungal	25 (0.11)	15 (0.15)	10 (0.08)	0.20
Polymicrobial[6]	153 (0.37)	63 (0.37)	90 (0.38)	0.56

Footnote:

[1]P value using Mantel-Haenszel test adjusted for center

[2]Data on total admission were available per month. Therefore, infants admitted before the pandemic who had infection episodes in pandemic period or had infections both before and during the pandemic were excluded from this table. This includes six infants with viral infections and 51 infants with 104 bacterial/fungal infections.

[3]Infants can be included under more than one source of infection and under more than one pathogen group if they had more than one source of infection or infection with more than one pathogen group. For example, if an infant had both BSI and UTI they would be included under both sources of infection.
[4]BSI or meningitis includes infants with these infection sources, with or without concomitant UTI. UTI alone includes infants whose UTI was not associated with any other source of infection.
[5]CLABSI information missing for six BSI events.
[6]If more than one organism is isolated from the same specimen on the same day, it was considered a polymicrobial infection, and the proportion of such infections is shown. Organisms in these events were not included in the pathogen group calculations. A list of all organisms, including those identified in polymicrobial infections, can be found in eTable 8.
Abbreviations: BSI – bloodstream infection, CLABSI – central line-associated bloodstream infection, CoNS – coagulase-negative staphylococci, HAI – healthcare associated infection, UTI – urinary tract infection.

eTable 6: Estimates (95% confidence limits) of changes in HAI rates per 1000 patient-days from the time series analyses

**Outcome**[1] estimate (95% CI)	**Intercept**	**Pre-pandemic trend**	**Change in level at start of pandemic**[2]	**Pandemic trend**	**Change in trend**
**Viral infection rate per 1000 PD**	-1.40 (-1.75, 1.05)	0.010 (-0.014, 0.034)	-1.41 (-2.13, -0.69)	0.038 (0.009, 0.067)	0.028 (-0.009, 0.065)
***P* value**	<0.001	0.408	<0.001	0.011	0.138
**Bacterial/fungal infection rate per 1000 PD**	0.50 (0.35, 0.64)	0.004 (-0.005, 0.014)	-0.16 (-0.35, 0.00)	0.009 (0.001, 0.017)	0.005 (-0.007, 0.017)
***P* value**	<0.001	0.381	0.082	0.023	0.445

Footnote: [1]Models use lag term to induce correlations among rates over time. [2]Pandemic start defined in study as 4/1/2020.
Abbreviations: LOS – Late onset sepsis, CONS - coagulase negative staphylococci, PD – patient days, CLABSI – central line associated bloodstream infection

eTable 7: Rate ratios of viral HAI the first and second year of the pandemic compared to corresponding periods before the pandemic by seasons

Before pandemic	Pandemic period	Adjusted rate ratio	95% CI
**First year of the pandemic**			
Apr 2018– Sep 2018, and Apr 2019– Sep 2019	Apr 2020 - Sep 2020	0.15	0.07, 0.35
Oct 2018– Mar 2019, and Oct 2019 – Mar 2020	Oct 2020 – Mar 2021	0.40	0.23, 070
**Second year of the pandemic**			
Apr 2018– Sep 2018, and Apr 2019– Sep 2019	Apr 2021 - Sep 2021	0.45	0.27, 0.74
Oct 2018– Mar 2019, and Oct 2019 – Mar 2020	Oct 2021 – Mar 2022	0.58	0.36, 0.92

eTable 8: Organism distribution in bacterial/fungal HAI before and during the pandemic

**Organisms**[1]<br>n (column %)	**BSI and/or meningitis**[2]			**Treated UTI**[3]		
	**Total**	**Before pandemic**	**During pandemic**	**Total**	**Before pandemic**	**During pandemic**
	**1175**	**558**	**617**	**1070**	**466**	**604**
Gram-positive	853 (72.6)	398 (71.3)	455 (73.7)	320 (29.9)	143 (30.7)	177 (29.3)
Coagulase-negative staphylococcus	528 (44.9)	266 (47.7)	262 (42.5)	58 (5.4)	34 (7.3)	24 (4.0)
Staphylococcus aureus	173 (14.7)	76 (13.6)	97 (15.7)	25 (2.3)	10 (2.1)	15 (2.5)
Enterococcus sp.	58 (4.9)	17 (3.0)	41 (6.6)	218 (20.4)	88 (18.9)	130 (21.5)
Streptococcus agalactiae	56 (4.8)	22 (3.9)	34 (5.5)	9 (0.8)	5 (1.1)	4 (0.7)
Streptococcus pneumoniae	1 (0.1)	0 (0.0)	1 (0.2)	0 (0)	0 (0.0)	0 (0.0)
Viridians/other streptococci	21 (1.8)	11 (2.0)	10 (1.6)	8 (0.7)	4 (0.9)	4 (0.7)
Other	16 (1.4)	6 (1.1)	10 (1.6)	2 (0.2)	2 (0.4)	0 (0.0)
Gram-negative	300 (25.5)	151 (27.1)	149 (24.1)	720 (67.3)	306 (65.7)	414 (68.5)
Escherichia coli	145 (12.3)	76 (13.6)	69 (11.2)	205 (19.2)	91 (19.5)	114 (18.9)
Klebsiella sp	71 (6)	33 (5.9)	38 (6.2)	273 (25.5)	114 (24.5)	159 (26.3)
Enterobacter sp	25 (2.1)	15 (2.7)	10 (1.6)	92 (8.6)	45 (9.7)	47 (7.8)
Pseudomonas aeruginosa	22 (1.9)	7 (1.3)	15 (2.4)	36 (3.4)	15 (3.2)	21 (3.5)
Serratia sp.	18 (1.5)	9 (1.6)	9 (1.5)	56 (5.2)	20 (4.3)	36 (6.0)
Acinetobacter sp.	5 (0.4)	4 (0.7)	1 (0.2)	1 (0.1)	0 (0.0)	1 (0.2)
Morganella morganii	3 (0.3)	1 (0.2)	2 (0.3)	5 (0.5)	1 (0.2)	4 (0.7)
Other	11 (0.9)	6 (1.1)	5 (0.8)	52 (4.9)	20 (4.3)	32 (5.3)
Fungus	22 (1.9)	9 (1.6)	13 (2.1)	30 (2.8)	17 (3.6)	13 (2.2)
Candida albicans	10 (0.9)	4 (0.7)	6 (1.0)	14 (1.3)	10 (2.1)	4 (0.7)
Non-albicans candida	11 (0.9)	5 (0.9)	6 (1.0)	9 (0.8)	5 (1.1)	4 (0.7)
Exophiala species	1 (0.1)	0 (0.0)	1 (0.2)	0 (0)	0 (0.0)	0 (0.0)
Yeast	-	-	-	7 (0.7)	2 (0.4)	5 (0.8)

Footnotes:

[1]Organisms identified in cases of polymicrobial infection, i.e. more than one organism identified from the same source, are included under the respective pathogen categories. Therefore, the total number of organisms does not add up to the total HAI episodes. Of the 1086 total BSI and/or meningitis episodes, 79 (7.3%) were polymicrobial: 33/520 (6.3%) episodes before and 46/566 (8.1%) during the pandemic. Of the 883 treated UTI episodes, 172 (19.5%) were polymicrobial: 67/392 (17.%) before and 105/491 (21.4%) during the pandemic.
[2]Any BSI and/or meningitis episodes are included. There were 58 BSI cases where urine was also positive for the pathogen.
[3]Includes UTI episodes where only urine was positive and met criteria for treated UTI.

eTable 9: Bacterial/fungal HAI incidence rates per 1000 patient-days among extremely low birth weight infants

**Characteristics,** *No. of HAI episodes (rate per 1000 PD) unless otherwise indicated*	**Total**	**Before pandemic**	**During pandemic**	**Adjusted Rate Ratio[1]**	**95% CI**	**P**
**Patient days (12 sites), n**	366,733	176,173	190,560	--	--	
Bacterial/fungal infection events	1055 (2.88)	482 (2.74)	573 (3.01)	1.1	0.98, 1.25	0.11
Type of infections[3]						
Any BSI	618 (1.69)	294 (1.67)	324 (1.70)	1.03	0.88, 1.20	0.76
Any Meningitis	20 (0.06)	14 (0.08)	6 (0.03)	0.4	0.15, 1.03	0.06
BSI and/or meningitis	632 (1.72)	306 (1.74)	326 (1.17)	0.99	0.85, 1.16	0.91
UTI (treated) alone	423 (1.15)	176 (1.0)	247 (1.3)	1.3	1.07,1.58	0.01
UTI (confirmed) alone	252 (0.69)	109 (0.62)	143 (0.75)	1.22	0.95, 1.56	0.12
CLABSI, n (per 1000 line-days)[4]	159 (1.73)	71 (1.51)	88 (1.96)	1.32	0.96, 1.80	0.09
BSI and/or meningitis by pathogen group						
CONS	281 (0.77)	140 (0.80)	141 (0.74)	0.93	0.74, 1.18	0.54
Gram-positive (excluding CONS)	146 (0.40)	59 (0.34)	87 (0.46)	1.37	0.99, 1.91	0.06
Gram-negative	136 (0.37)	74 (0.42)	62 (0.33)	0.78	0.56, 1.10	0.16
Fungal	16 (0.04)	7 (0.04)	9 (0.05)	1.22	045, 3.28	0.69
Polymicrobial[5]	53 (0.15)	26 (0.15)	27 (0.14)	0.98	0.57, 1.67	0.93
Treated UTI by pathogen						
CONS	17 (0.05)	6 (0.03)	11 (0.06)	1.73	0.64, 4.69	0.28
Gram-positive (excluding CONS)	69 (0.19)	26 (0.15)	43 (0.23)	1.54	0.95, 2.51	0.08
Gram-negative	242 (0.66)	112 (0.64)	130 (0.68)	1.07	0.83, 1.38	0.58
Fungal	13 (0.04)	6 (0.03)	7 (0.04)	1.08	0.36, 3.23	0.88
Polymicrobial[5]	82 (0.22)	26 (0.15)	56 (0.29)	1.99	1.56, 3.17	0.004
Confirmed UTI by pathogen						
CONS	13 (0.04)	6 (0.03)	7 (0.04)	1.11	0.37, 3.29	0.86
Gram-positive (excluding CONS)	47 (0.13)	19 (0.11)	28 (0.15)	1.36	0.76. 2.44	0.30
Gram-negative	185 (0.51)	81 (0.46)	104 (0.55)	1.19	0.89, 1.59	0.24
Fungal	7 (0.02)	3 (0.02)	4 (0.02)	1.25	0.28, 5.57	0.77

Footnote:

[1]Pandemic to pre-pandemic rate ratio calculation is adjusted for center. Rate change is significant if the CI for the ratio does not include one.
[3]Any BSI and any meningitis includes events of BSI or meningitis (with or without concomitant infection with another source).
[4]CLABSI information missing for 6 BSI events. Number of line days = 46,901 (pre) and 45,003 (post)
[5]If more than one organism is isolated from the same specimen on the same day it was considered a polymicrobial infection and the proportion of such infections is shown. Organisms in these events were not included in the pathogen group calculations.
Abbreviations: BSI – bloodstream infection, CLABSI – central line-associated bloodstream infection, CoNS – coagulase-negative staphylococci, HAI – healthcare associated infection, UTI – urinary tract infection, PD – patient days
