# Supplementary material for: Health Care–Associated Infections Among Neonates During the COVID-19 Pandemic
Source: JAMA Netw Open. 2026 Jan 28;9(1):e2555623. doi: 10.1001/jamanetworkopen.2025.55623 (PMC12853212; doi:10.1001/jamanetworkopen.2025.55623)
Supplement: Supplement 2. — Data Sharing Statement [file jamanetwopen-e2555623-s002.pdf]

Data Sharing Statement

Mukhopadhyay. Health Care–Associated Infections Among Neonates During the COVID-19 Pandemic. *JAMA Netw Open*. Published January 28, 2026.
doi:10.1001/jamanetworkopen.2025.55623

Data
Data available: Yes
Data types: Data dictionary, Deidentified participant data
How to access data: Data sharing available after appropriate data use agreement. Contact mukhopadhs@chop.edu
When available: With publication

Supporting Documents
Document types: None

Additional Information
Who can access the data: Researchers whose proposed data request has been approved by PI co-investigators from other sites and with appropriate data use agreement with CHOP
Types of analyses: Epidemiological studies
Mechanisms of data availability: Data can be made available if required approvals are obtained via a CHOP based secure share platform.
